# Vancomycin Hypersensitivity Diagnosed by Lymphocyte Blast Transformation

**DOI:** 10.1155/2011/562620

**Published:** 2011-11-24

**Authors:** Nguyen P. Tran, Jeremy Katcher, Erin Rohman, Mary Francis Hall, Christie F. Michael, Isao Miyairi, D. Betty Lew

**Affiliations:** ^1^Allergy/Immunology, Le Bonheur Children's Hospital, Memphis, TN 38103, USA; ^2^Diagnostic Immunology, Memphis Pathology Laboratories, Memphis, TN 38141, USA

## Abstract

A 15-year-old male admitted for Pott's puffy tumor developed recurrent episodes of fever, diffuse morbilliform rash, eosinophilia, and tubulointerstitial nephritis while on multiple antibiotics. Lymphocyte blast transformation (LBT), a method of detecting cellular immune response by measuring levels of interferon-**γ** (IFN-**γ**), was used to diagnose vancomycin hypersensitivity and guide antibiotic selection.

## 1. Introduction

 Drug allergies have a significant impact on patient morbidity and overall cost of the practice of medicine. Anywhere from 10–20% of hospitalized patients will have an adverse drug reaction [1]. The most common clinical presentation of these reactions is cutaneous, such as urticaria and maculopapular eruptions [2]. Adverse drug reactions from vancomycin specifically can range from red man syndrome to leukocytoclastic vasculitis to anaphylaxis [3]. Less common reactions include exfoliative dermatitis, linear IgA bullous dermatosis, and delayed hypersensitivity reaction [4, 5].

 Currently, diagnostic methods for suspected allergic reaction to vancomycin are limited, and the diagnosis is often made based by history alone. Skin puncture testing with vancomycin results in frequent false positives secondary to direct degranulation of mast cells [6]. Polk et al. performed vancomycin skin testing on healthy volunteers and found that all subjects tested had a positive reaction at concentrations ≥10 *μ*g/mL [7].

 We report a case of an adolescent patient who developed a morbilliform rash while on multiple antibiotics. Lymphocyte blast transformation (LBT) was performed to involve antibiotics to help determine the culprit. Although T-cell Rx test (CD69 upregulation) for vancomycin delayed hypersensitivity has become recently available [8], vancomycin hypersensitivity documented by LBT has not been previously reported.

## 2. Case Report

A 15-year-old male with a history of craniosynostosis and developmental delay presented to the Emergency Department with fever, headache, and swelling of the left forehead. A CT scan revealed left frontal sinusitis with extension of the inflammatory process through the left frontal bone into the subperiosteal region (Pott's puffy tumor) and subgaleal region. The patient also had evidence of metallic sutures and fenestrated plate to the superior margin of the left frontal sinus consistent with his past medical history of repaired craniosynostosis at 6 months of age. Family history and social history were unremarkable for drug reactions. The patient was started on vancomycin and ceftriaxone upon admission and underwent bifrontal craniotomy, exoneration of frontal sinuses with vascularized pericranial rotational graft, an endoscopic left-sided ethmoidectomy, and maxillary antrostomy. Gram stain of the purulent material obtained from the surgery revealed Gram-positive cocci in pairs, chains, and clusters while the culture grew rare anaerobic Gram-negative cocci, which we were unable to speciate. Given concerns of polymicrobial infection with potential foreign body involvement, his regimen was changed to vancomycin and meropenem. The patient defervesced, and his edema subsided. On hospital day 8–10, he developed a diffuse, erythematous morbilliform rash, fevers up to 40°C, and elevated blood pressures with systolic measurements to 160 mmHg. Laboratory examination revealed new onset eosinophilia (max. absolute eosinophil count 980/mm^3^), mild elevation of creatinine and 1−2+ proteinuria, consistent with drug associated hypersensitivity and tubulointerstitial nephritis. Vancomycin and meropenem were discontinued and changed to ceftriaxone and metronidazole on hospital day 10. The patient promptly defervesced with gradual improvement of the rash, but on hospital day 14 developed low-grade fevers (T_m_ 38.1°C) and recurrence of diffuse rash. Skin exam revealed widespread maculopapular erythematous rash, prominent on the arms, legs, and trunk, especially abdomen. No blisters or drainage were associated with the rash and there was no mucosal involvement. 

LBT was performed as described in the methods and results section. Lymphocytes incubated with vancomycin showed a robust response and those incubated with metronidazole showed an equivocal response (likely contributing to the recurrence of rash on hospital day 14). The remainder of the antibiotics tested did not show significant IFN-*γ* production. The patient tolerated a treatment regimen of ceftriaxone and clindamycin with resolution of rash and return to his baseline temperatures. He completed a total course of 4-weeks of antibiotics without any signs of relapse.

## 3. Methods and Results

 Lymphocyte blast transformation is a method for detection of cellular immune response based upon the premise that stimulated or sensitized lymphocytes will revert to blast formation and produce detectable levels of IFN-*γ*, which are directly proportional to the level of sensitization. Testing was performed using the QuantiFERON-CMI testing kit (Cellestis, Victoria, Australia, http://www.cellestis.com/) [9]. Heparinized whole blood was collected, and *ex vivo* LBT was performed with phytohemagglutinin (PHA), pokeweed mitogen (PWM), concanavalin A (Con A) to evaluate the patient's baseline immune function. The patient's whole blood was incubated with antibiotics at varying dilutions designed to mimic actual serum concentration for 18 hours at 37°C in a 24-well tissue culture plate. Then using a pipette, 200–300 *μ*L of plasma above the sedimented red cells was transferred to a new well plate (anti-human IFN-*γ* antibodies bound to the solid phase), and IFN-*γ* production was then quantitatively measured using enzyme-linked immunosorbent assay (ELISA) utilizing enzyme-labeled IFN-*γ* antibodies in solution. The interferon cutoff was set at 0.16 IU/mL, which was determined by plotting the optical density against a standard curve generated according to the manufacture's instructions.

**Figure 1:**
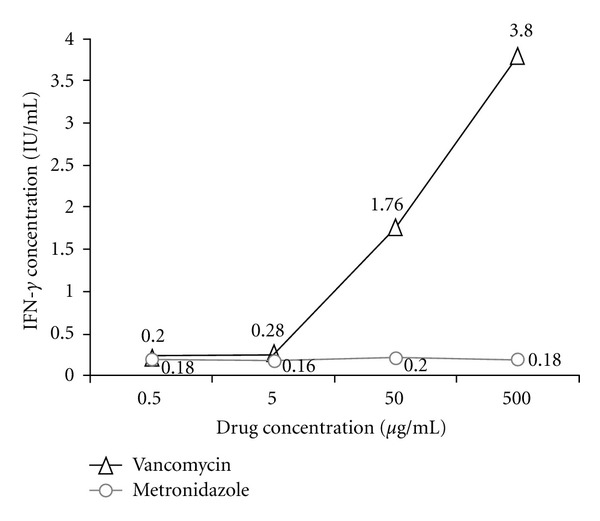
Secreted fraction of INF-*γ* production by the patient's peripheral lymphocytes after 18 h stimulation with various concentrations of Vancomycin and Metronidazole. The patient did not produce any detectable levels of INF-*γ* with Ceftriaxone, Amoxicillin, and Meropenem stimulation at all drug concentrations (<0.16 IU/mL). Likewise, the levels of INF-*γ* production in Vancomycin-naïve control subject were below the threshold of detection (<0.16 IU/mL) at all Vancomycin concentrations.

 In our patient, his response to PWM, PHA, and the kit's mitogen positive control was within the expected ranges. The positive control included in the QuantiFERON-CMI testing kit generally elicits the greatest IFN-*γ* response for any given individual. The response to Con A was less than expected at 0.269 IU/mL (effective response >0.6). There was a remarkable concentration-dependant response to vancomycin, an equivocal response to metronidazole, and no response to ceftriaxone, amoxicillin, and meropenem; see Figure 1. Vancomycin hypersensitivity was diagnosed.

## 4. Discussion

 Maculopapular eruptions are a common manifestation of nonimmediate allergic drug reactions and are mediated via drug-specific T cells. Drugs are low-molecular-weight chemicals that can interact with T cells, specifically their receptors, to activate an immune response. Once activated, the drug-specific T cells migrate into the skin and cause inflammatory damage with the release of various cytokines [10].

 One of the original methods of determining cell stimulation was via the lymphocyte transformation test, which is an *in vitro *assay that measures the rate of ^3^H-thymidine or bromodeoxyuridine uptake in replicating DNA [11]. Its clinical use has been discussed in the literature for detection of delayed hypersensitivity reactions to certain antibiotics, antiepileptics, antihypertensives, NSAIDS, anesthetics, and radiocontrast media [12]. LBT has the advantage over the lymphocyte transformation test of a quicker turn-around time (usually 24–48 hours) since there is no radioisotope or need to culture cells. Recently, Beeler et al. reported on the use of CD69 upregulation on T cells as an activation marker for delayed-type drug hypersensitivity [8]. In contrast, LBT is a functional assay for detecting IFN-*γ* production by sensitized T cells in response to specific antigen or mitogen [9]. There is no previously published report on the use of increased IFN-*γ* production to diagnose vancomycin hypersensitivity.

 The patient described in this paper demonstrates a common scenario encountered by consulting allergists and infectious diseases specialists. Due to the lack of specificity and sensitivity of skin testing for some medications, the patient history is often relied upon to determine which antibiotic is the causative agent in a drug reaction. Reliable diagnosis, though difficult, is necessary for safe and effective patient treatment. This patient had a complicated sinus infection requiring long-term broad-spectrum coverage, which was further complicated by recurrent episodes of fever and rash resulting in the use of multiple classes of antibiotics. His morbilliform rash was initially thought to be secondary to cephalosporins. However, we were able to determine that vancomycin and possibly metronidazole were likely to be responsible for the patient's fever and rash. In this case, LBT testing guided us in establishing an alternative regimen of ceftriaxone and clindamycin. 

The marked INF-*γ* levels detected using the quantiFERON-CMI kit indicates the presence of a specific T-cell-mediated immune response against vancomycin, which is the immunologic basis for delayed-type hypersensitivity. Vancomycin concentrations used were within therapeutic levels and combined with the patient's clinical course indicate that LBT was useful in the diagnosis of vancomycin hypersensitivity in this patient. Since it is a cellular immune response, unsensitized individuals should show zero production of interferon. Clearly, larger studies are needed to determine its utility in various clinical scenarios. There was a mild response to metronidazole, which correlates with the relatively milder clinical manifestation observed during metronidazole therapy and possible metronidazole hypersensitivity. The results of a negative response to *β*-lactam agents were interpreted with caution, and clinical response was monitored.

A potential limitation of LBT would be non-discriminatory IFN-*γ* production that may be seen in a scenario of multidrug hypersensitivity induced by superantigens. However, even in such a case, negative results can still be helpful. Results need to be reproduced in a larger sample size. Further evaluation is also needed to see if it can be used for hypersensitivity to other medications and if other cytokines may be more sensitive or specific for drug-related delayed-type hypersensitivity.

## 5. Conclusions

Lymphoblast transformation is a useful test in confirming the etiology of vancomycin-induced delayed hypersensitivity reaction.
